# Program Practices Predict Intergenerational Interaction among Youth and Older Adults

**DOI:** 10.1093/geroni/igab046.2910

**Published:** 2021-12-17

**Authors:** Shannon Jarrott, Shelbie Turner, Jill Juris Naar, Rachel Scrivano, Raven Weaver

**Affiliations:** 1 The Ohio State University, Columbus, Ohio, United States; 2 Oregon State University, Oregon State University, Oregon, United States; 3 Appalachian State University, Boone, North Carolina, United States; 4 Washington State University, Pullman, Washington, United States

## Abstract

Non-familial intergenerational programs engage younger and older people in shared programming for mutual benefit, frequently involving senior centers or adult day programs and preschools. With growing interest in the potential benefits of intergenerational strategies, it is imperative to know their effects on participant interaction during intergenerational programming. To address this knowledge gap, activity leaders at five sites serving older adults and/or preschoolers received training to implement 14 evidence-based practices during intergenerational activities involving 109 older adult and 105 preschool participants over four years. We utilized multi-level modeling to test whether variations in implementation of practices were associated with variations in participants’ responses to programming on a session-by-session basis. For both preschool and older adult participants, analyses revealed that the implementation of certain practices was associated with significantly more intergenerational interaction. Specifically, when person-centered best practices (e.g., leading activities that are age- and role-appropriate for older adults) were implemented, preschoolers (estimate=5.83, SD=2.11, p=0.01 and older adults (estimate=5.11, SD=.10, p=0.02) had more intergenerational interaction. Likewise, when environmental-centered best practices were implemented, such as pairing materials between intergenerational partners, preschoolers (estimate=6.05, SD=1.57, p=0.002) and older adults (estimate=6.50, SD=1.85, p=0.001) had more intergenerational interaction. Our findings reveal session-by-session variation in intergenerational interaction that can be impacted by implementation practices, which highlights the importance of training activity leaders to implement evidence-based practices. Researchers and practitioners should consider how session-by-session variation in program implementation affects participant response.

